# Urban Spillover, Health Disparities, and Community Strengths in Small Towns in Central Texas

**DOI:** 10.3390/healthcare13050501

**Published:** 2025-02-26

**Authors:** Ya-Ching Huang, Valerie Zapien, Yanyan Chen, Sara Le, Ashton Cumberbatch, Carmen Valdez

**Affiliations:** 1 College of Nursing, Texas A&M University, 3950 North A.W, Grimes Boulevard, Round Rock, TX 78665, USA; 2 Willamson County and Cities Health District, Round Rock, TX 78664, USA; Valerie.Zapien@wilcotx.gov; 3 School of Public Health, Texas A&M University, College Station, TX 77843, USA; chen2022@exchange.tamu.edu; 4 Department of Kinesiology and Health Education, University of Texas at Austin, Austin, TX 78712, USA; sal4393@my.utexas.edu; 5 Equidad ATX, Austin, TX 78721, USA; ashton@equidadatx.org; 6 UT School of Public Health San Antonio, UT Health San Antonio, San Antonio, TX 78229, USA; valdezcr@uthscsa.edu

**Keywords:** small towns, social determinants of health, health disparities

## Abstract

**Background**: Small-town residents face healthcare inequities that result in poorer outcomes compared to those living in urban areas. These disparities are deeply rooted in socioeconomic and demographic factors as well as in access to healthcare services. This study aimed to explore health disparities and community strengths within small towns in Williamson County, Central Texas, intensified by urban spillover. **Methods**: A qualitative study was designed using the Social Determinants of Health Model as the conceptual framework. We conducted eight in-depth interviews with local stakeholders. Each interview lasted 60–90 min and was audio-recorded, transcribed verbatim, and subjected to thematic analysis by trained researchers. **Results**: The participants reported that significant population growth, via individuals drawn from the rapidly expanding cities in proximity to their small towns, dramatically impacts lives. Eight main themes emerged: (1) low levels of community and professional health literacy and a lack of healthcare navigation support in schools and community-based support systems; (2) limited funding for healthcare services and limited capacity to support access to them; (3) limited access to food that can support healthy dietary patterns; (4) inadequate transportation services; (5) substance use, violence, and crime; (6) stressful community dynamics and an environment that negatively influences individual and community well-being; (7) financial instability and unreliable and insufficient infrastructure; and (8) community strengths and potential resources. **Conclusions**: Our findings reveal critical healthcare disparities in these small towns. Harnessing funding support and community unity is vital for developing equitable healthcare solutions and improving the well-being of residents in these rapidly evolving locales.

## 1. Introduction

The term ‘small town’ typically refers to a community with a relatively small population, often less than 5000 people [1]. These small towns are characterized by close-knit social groups that foster a sense of community and belonging. However, small towns impacted by urban spillover face several challenges concerning their sense of community and belonging, including rapid population growth that strains local resources and infrastructure, economic shifts that alter the local economy, and an increased demand for services like schools and healthcare. Additionally, the influx of new and culturally diverse residents (i.e., aging populations, families with young children and teenagers, and different races, ethnicities, and languages) can change the social dynamics, making it difficult to maintain a sense of community. Here, social dynamics refers to the interconnected and interdependent nature of how a town functions through its relationships, values, institutions, and norms to form a cohesive whole [2]. These impacts could include both positive development and significant challenges for residents in these small towns. The purpose of this article is to explore health disparities and community strengths in geographically isolated small towns affected by urban spillover.

The classification of where one lives should not be a determining factor for their qualitative health status, but it is, for across hundreds of small towns and farms today, we see a population that tends to be older, with greater health disparities and often lacking both access to care as well as financial resources to address their health concerns [3]. The Health Resources and Services Administration (HRSA) aims to provide equitable health care to people who are geographically isolated and economically or medically vulnerable [4]. However, some small towns that are not classified as “rural” by the USA Census Bureau [5] lack critical healthcare resources and are not eligible to apply for rural health grants [6]. Additionally, while the HRSA website provides data on provider shortage areas to highlight rural communities needing better access to primary care, dental, and mental health services [7], these same small towns often do not qualify as shortage or medically underserved areas.

### 1.1. Challenges Regarding Health Disparities Between Small Towns and Rural Areas

According to the 2020 Census, small towns with populations under 5000 have experienced uneven growth across different U.S. regions. While small towns in the Northeast and Midwest have experienced a decline, those in the South and West have experienced significant population growth, increasing by 6.7% and 13.3%, respectively [1]. As of 2024, 9.2% of the population of Williamson County lived in “small towns”, defined as an area with fewer than 2000 housing units and less than 5000 people [8]. Small, geographically isolated towns face health disparity challenges similar to those affecting rural areas. These health disparities arise from several interconnected factors. Limited healthcare facilities and specialist availability necessitate long-distance travel for care [9], while economic barriers, such as high rates of uninsured individuals and financially strained local healthcare providers, exacerbate access issues [10]. Workforce shortages, including difficulties in recruiting and retaining healthcare professionals, further strain the healthcare system [11]. Additionally, demographic shifts have been observed with the growth of small towns. For instance, small towns are becoming more ethno-racially diverse, driven in part by Hispanic population growth [12], especially in southern states. These demographic changes may increase barriers to quality healthcare due to difficulties in finding culturally competent healthcare providers [13] or limited organizational support for recruiting health professionals across diverse racial/ethnic backgrounds [14].

### 1.2. Williamson County, Texas, Population Change Projections and Background

Williamson County, situated north of Austin and part of the Austin–Round Rock–San Marcos metropolitan statistical area, is centrally located in Texas, lying just north of the State Capital. This county has experienced dramatic population growth. As of 2023, Williamson County was the third fastest growing county in Texas, with a 162.9% growth rate [15]. The population of Williamson County is estimated to reach 1.6 million by 2050, more than double what it is now [16].

According to the 2022 Williamson County Community Health Assessment, among the age groups, only the “65+” population is projected to increase, growing from 12.9% to 18.1%. The percentage of the population that is Hispanic/Latino is projected to increase from 24.4% to 27.1% by 2050, while the percentage of the population that is Asian American is projected to triple, increasing from 6.8% to 25.5%. The percentage of the population that is White Non-Hispanic is projected to decrease from 59.4% to 36.3% [17]. This rapid county-wide increase in population and economic expansion have significantly strained existing infrastructure, including in regard to broadband internet accessibility [18], healthcare resources [19], housing demand [20], parks/trails, and the natural environment [21].

Based on the Housing Affordability Challenge Report [22], Texas’s median home prices rose by 40 percent between 2019 and 2023. After a period of rapidly rising inflation rates, mortgage rates rose to a 23-year high of 7.79 percent in October 2023. A rapid upswing in prices in Texas and the USA began in 2020 due to an increase in opportunities to telework, federal stimuli and unemployment payments, low mortgage rates, a surging stock market, and accelerating domestic migration to this state. Texas’s price growth was more aligned with fundamentals, including employment, income, new-home construction, and population growth. Middle-class and low-income families wanting to own a home had to move farther outside city centers where jobs were located, increasing commute times and traffic, resulting in urban spillover. Urban spillover refers to the effects that urban development and activities have on surrounding areas, often extending beyond a city’s boundaries. These effects can be economic, social, or environmental. Many Williamsons County residents commute to Travis County and other surrounding counties for work. From 2017 to 2022, 43.4% of Williamson County residents were recorded as commuting to work (travelling for more than 30 min); in comparison, in the rest of Texas, 39% of residents commute to work [23].

### 1.3. Community Health Assessments and Health Equity Zones

**Figure 1:**
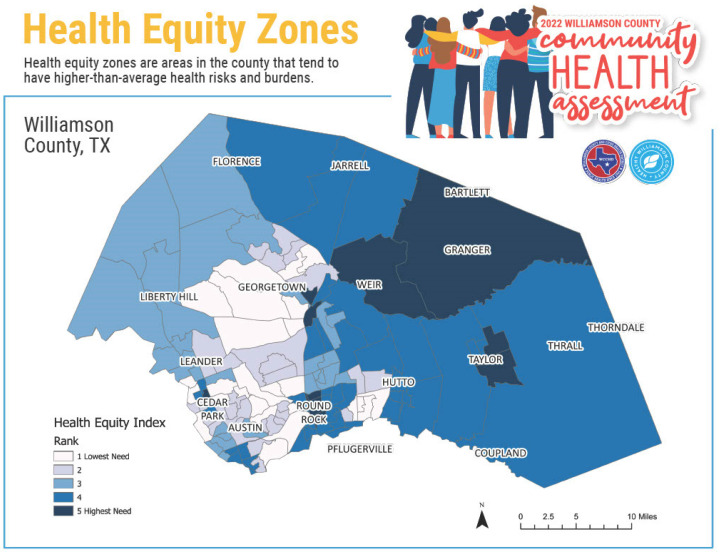
Health equity zones.

Health equity is defined as a state wherein everyone has the same opportunity to live the healthiest life possible, no matter who they are, where they live, or how much money they make [24]. The 2024 Health Equity Index is a measure of socioeconomic need that is correlated with poor health outcomes. All zip codes, counties, and county equivalents in the United States are given an index value and ranked from 1 (low need) to 5 (high need) based on their index value relative to similar locations within the region [25]. According to the Health Equity Zone Index, although Williamson County has been ranked as one of the healthiest communities in Texas since 2010 for overall health outcomes and second in terms of health factors, several zip codes in Williamson County, particularly in more geographically isolated small towns, have been designated as Health Equity Zones (HEZs). Communities with the highest index values are estimated to have the highest socioeconomic needs correlated with poor health outcomes [25]. HEZs in Williamson County include Bartlett, Coupland, Granger, Jarrell, Weir, Taylor, Thrall, and specific zip codes within larger cities such as Georgetown, Round Rock, and Cedar Park (Figure 1) [17]. The differences between the inequities of small towns and urban communities highlight the importance of understanding the needs of community residents at the zip-code level to access healthcare and other services, achieve better overall wellbeing, and create solutions that better meet their needs.

### 1.4. Social Determinants of Health Framework

Applying a Social Determinants of Health (SDOH) Framework, in the present article, we explore (a) non-medical factors (e.g., education access and quality, transportation, and economic stability) that influence health in small towns in Williamson County, (b) the groups most affected by health disparities, and (c) the strengths within these communities that can be leveraged to reduce disparities [26]. The findings from this study will inform future projects aimed at developing tailored interventions to improve health outcomes in these areas and can supplement community health assessments and health improvement plans devised by local health departments, which often do not include small towns.

## 2. Materials and Methods

### 2.1. Research Design and Informants

A thematic qualitative study design was conducted and analyzed using deductive analytical methods, with a focus on thematic analysis [27] based on the SDOH framework. Thematic analysis is a technique for systematically describing and interpreting qualitative data by assigning codes and then organizing these codes into themes. After this, the identified themes are analyzed and presented. This method blends a structured approach with a researcher’s subjective interpretation, enabling systematic documentation of each step in the data analysis process while also providing the researcher with the creative freedom to derive meaning from the data [28].

After the study protocol was approved by the University’s Institutional Review Board (IRB number: STUDY2024-0126), purposive sampling methods were employed to recruit potential informants from five small towns in Williamson County. This qualitative study was conducted by recruiting informants from referrals from existing collaborative relationships through the Healthy Williamson County (HWC) Coalition of the Williamson County and Cities Health District (WCCHD). Coalition partners were asked to identify potential key informants based on their leadership roles and experience working directly with residents who are medically underserved, have low incomes, and are part of a minority population, specifically in small towns with populations of less than 2000 and identified HEZs ranked 3–5. Based on this criterion, five small towns were identified: Bartlett, Coupland, Granger, Jarrell, and Thrall. In addition, in these small towns, less than 1% of the residents participate in WCCHD’s periodic Community Health Assessment.

After potential informants were identified by the HWC Coalition members, further screening inclusion criteria were applied: the participants had to (1) be at least 18 years old; (2) be able to communicate in English; and (3) have access to the Internet and appropriate device(s) to attend the online meeting. The HWC Coalition Coordinator contacted and recruited key informants via email to explain this study’s purpose. After confirming a potential informant’s eligibility, the research team invited them to an individual interview that took place in April 2024, 3–15 days after first contact was established.

Key informants included leaders and staff representatives from K-12 schools, city and county officials, and members of community-based organizations. Twelve informants were identified, ten of whom agreed to participate; however, only eight were interviewed, as two informants could not attend the scheduled interviews due to unanticipated workplace conflicts and were not available for a rescheduled interview.

### 2.2. Data Collection

The guided interview questions were created using a modified version of those published in the Mobilizing for Action through Planning and Partnerships 2.0 Framework [29]. The interview questions were reviewed and approved by the Coalition’s Community Advisory Board, which consists of community members with lived experience related to health disparities (Table 1). The research questions were “How do critical health issues specifically impact low-income, underserved, or uninsured populations?” and “What are some of the community’s greatest strengths and assets?”.

Before the interviews, the researchers explained the purpose of and procedures that would be used in this study, obtained informed consent, and administered a brief demographic questionnaire. A semi-structed, in-depth interview with a 60–90-min duration was conducted with the individuals via Zoom (Version 6.0.0) by the first two authors listed. All interviews were recorded and transcribed by Zoom and later validated by researchers. The key informant interview guide and questions were reviewed by the first two listed authors and refined over several iterations. They covered critical health issues in the county, structural factors (SDOH) that contribute to critical health issues, community assets, strengths of the community, and possible solutions to these health issues.

### 2.3. Data Analysis

Data management and analysis were conducted using Microsoft Word and Excel 2024. After the second listed author verified the accuracy of the Zoom transcription against the audio interview recordings, the first two listed authors individually reviewed all the transcripts and then collaboratively generated a detailed codebook with clear definitions for each code. Then, the first two listed authors collaboratively generated initial qualitative data codes for the first two interviews to ensure complete agreement in coding. Then, the first two listed authors collaborated with the third listed author to code the third interview, ensuring agreement in coding. After this agreement in coding was reached between the three researchers, thus ensuring inter-coder reliability, the remaining five interviews were assigned to the first three listed authors, with each interview coded by at least two people individually. Regular meetings were held to discuss the discrepancies and reach a consensus on the 8 transcripts. After finishing this first-cycle coding of all eight interviews, the authors collaborated by collating the codes into potential themes and gathering relevant data for each theme based on the SDOH model. The first four listed authors reviewed and refined the codes and these themes to ensure they accurately represented the data and defined and named each theme clearly. The process was repeated several times until the first four listed authors agreed, and when information saturation was reached and there were no new themes identified, the informant recruitment and interview phase of the study was concluded. Training in thematic analysis was provided as part of a mentoring program led by the sixth author, in which the fifth author served as a community mentor to the first two authors.

### 2.4. Rigor

Lincoln and Guba’s four criteria were applied to ensure scientific rigor [30]. Credibility was established through detailed interviews, peer debriefing among researchers, and data saturation. For confirmability, in addition to peer debriefing, the researchers engaged in self-reflection by maintaining notes in journals. Dependability was achieved by repeatedly conducting coding checks until consensus was reached among researchers. Transferability was ensured by providing very detailed descriptions and clearly explaining the limitations of this study.

## 3. Findings

Eight informants completed the Qualtrics demographics and consent survey and participated in one-on-one in-depth interviews with the researchers. All respondents were female, with the majority reporting themselves as being middle-aged (87.5%), except for one informant who was more than 60-years-old (12.5%). Half of the respondents identified as White (50.0%), while two informants identified as Hispanic White (25.0%), and one informant identified as mixed race (12.5%). The informants held various job titles, including school-based program coordinators, including a nurse, a counselor, and a therapist (50.0%); a city staff member (25.5%); an emergency management staff member (25.0%); a food pantry staff member (25.0%); and library and resources hub staff (25%). Detailed demographic characteristics are presented in Table 2.

### 3.1. Themes

**Figure 2:**
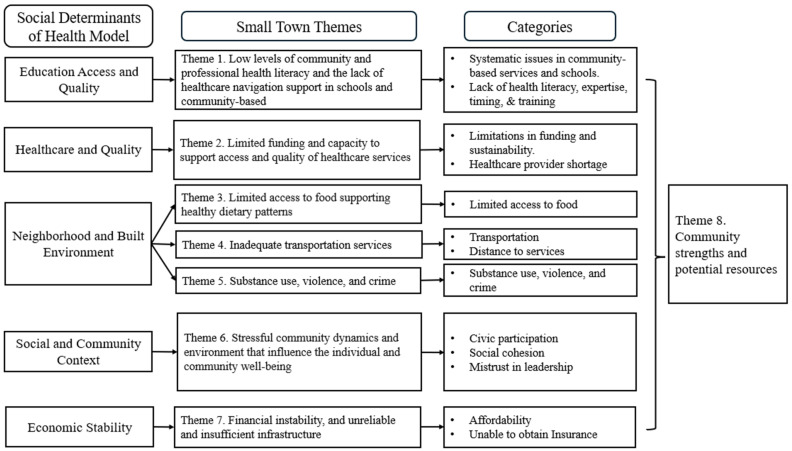
Thematic results.

Seven themes were formed from the focus group based on the SDOH framework and our findings. We categorized the themes as follows (Figure 2): Theme 1—low levels of community and professional health literacy and a lack of healthcare navigation support in schools and community-based support systems; Theme 2—limited funding for healthcare services and limited capacity to support access to them; Theme 3—limited access to food that can support healthy dietary patterns; Theme 4—inadequate transportation services; Theme 5—substance use, violence, and crime; Theme 6—stressful community dynamics and an environment that negatively influences individual and community well-being; and Theme 7—financial instability and unreliable and insufficient infrastructure.

An additional theme, Theme 8—community strengths and potential resources, was incorporated into the model to account for the positive and assertive connotations of residents living in small towns. The themes, categories, and frequency of the references to key codes are presented in Table 3.

### 3.2. Theme 1. Low Levels of Community and Professional Health Literacy and a Lack of Healthcare Navigation Support in Schools and Community-Based Support Systems

Systematic Issues. The informants repeatedly mentioned low levels of health education and literacy in their small towns, including in regard to residents not engaging in healthy behaviors, not understanding the resources available to the community, and not being fully aware of how to access the resources they knew about. The capacity (i.e., time and funding) of professionals to provide such education was limited. As education-focused institutions, small school districts typically do not have the resources required to hire multiple medically focused staff members to service the community. Therefore, often in small towns, non-medical school staff end up bearing the responsibility of helping to address the health needs of their students and families. School staff wear multiple hats or rely on outsourcing from community-based providers. The informants noted the difficulties related to finding providers/organizations willing to travel to them to provide these services for families and the general community. One informant said, “*We’re working to get more kinds of medical professionals to come to (our small town) but we can’t control that*”.

The informants also mentioned that changes in leadership cause shifts in priorities and funding, which sometimes results in important initiatives being dropped. A school may make progress in achieving specific objectives needed to impact health inequities only to be to be instructed by a new leader to pause or eliminate the initiative. To quote one informant, “*I just think that …sometimes in that change in leadership, just sometimes priorities change or funding changes*”.

Lack of health literacy, expertise, timing, and training. The informants repeatedly mentioned a pressing need to teach community members how to navigate healthcare due to health literacy issues and/or lacking the time needed to provide or access the resources needed. Moreover, professionals reported that referrals to external services often result in failed outcomes and negative feedback due to long wait times, limited eligibility criteria, lack of follow-ups, and difficulties in accessing services. The informants also reported concerns for families without internet or smartphone service struggling to navigate services that are available, making it overwhelming to find appropriate help when it is needed. An informant said, “*A lot of them don’t know how to navigate anything. There’s a lot of services, but they just don’t know how to get, you know, how or what to search or they don’t have internet, or they don’t have phone service*”.

According to the informants, although professionals are educated and trained in their profession, they too are often unaware of the various nuances and complexities involved in healthcare navigation. An informant explained that “*Navigating healthcare systems is challenging even for educated individuals who know the right questions to ask. It becomes even more difficult for those who are new to the system, new to the region, face language barriers, or are unfamiliar with technology*”.

Due to the need to manage auxiliary roles, staff from smaller schools reported having limited time to coordinate services that families need, like wraparound, follow-ups, and intensive case management support. An informant said, “*I just don’t think people recognize and maybe I’m sure people do not know how much initial support people need to sort of make these appointments and connect and get the right questions answered and even understand what’s being told to them. Like sometimes, we have to interpret, not even just language, but just like they don’t even understand what things even mean. You know, it feels like we (systems) make it really difficult*”.

Another informant mentioned that “*Parents dealing with a child’s mental health crisis find it overwhelming to navigate resources, determine insurance coverage, and access necessary services*”.

Small-school-district staff reported feeling overwhelmed by unmet needs of the families they serve. One reiterated: “*You imagine being a parent and saying ‘so your child has had this extreme mental health crisis. Here’s a list of resources. Go find someone to help them’. Do they take my insurance? And yeah, I mean it’s overwhelming for us. I can’t imagine how overwhelming it is for families*”.

### 3.3. Theme 2. Limited Funding for Healthcare Services and Limited Capacity to Support Access to Them 

Limitations regarding Funding and Sustainability. Limitations regarding funding and sustainability were a reported issue for school districts and community-based providers in small towns. Several healthcare services relevant to wellbeing in small towns rely on grant-funded projects, including for access to basic needs, e.g., mental health services and food. Funding for mental health services is in high demand for small-town schools, not only for students but for their families and the community at large. One informant reported, “*We don’t have a lot of access to these. We would connect them to a larger resource, such as Bluebonnet or Lone Star, or Central Counties. And then for the larger community as far as mental health, if your kid doesn’t attend the school, then you would have to travel somewhere else*”.

Another provided the following description: “*And in order to argue for funding. You’ve got to have data and in order to get data, you have to have participation and in order to get participation, you’ve got to make friends with people. And so that is certainly an area that I kind of pride myself in is knowing so many people in the community to be able to reach out and help you guys get responses for this type of stuff.”*

Healthcare provider shortage. The majority of the informants mentioned that there were many health-related resources, services, and providers in the county but that they are inaccessible to them. This results in residents going without the care or resources they need and may worsen their health statuses. Accessing any kind of care is a major challenge, with long wait lists and limited availability, especially for psychiatric services for children. In small towns, there are often no local doctors, dentists, or emergency services, with the nearest options being 15 miles away in surrounding urban or suburban areas. An informant said, “*Our town does not have a doctor, doesn’t have a dentist. It doesn’t have a minor emergency. The closest things that we have are miles away*”.

Another informant provided the following explanation: “*I would say that there’s sort of this overall vibe of like people are stretched very thin. People are wearing too many hats, people trying to do lots of things*”.

A shortage of providers means patients often do not receive adequate time with their healthcare professionals, leading to repeated health issues. An informant said, “*There are not as many providers to see those patients. The time spent is even less and so these patients are repeatedly having exacerbations and a lot of that stems from not being able to have that time with their provider, or with their nurse educator, so that education can be provided*”.

And another informant expressed, “*But just getting any kind of care is this major undertaking. And then, you know, just getting appointments right like the wait lists, the lack of oh, my gosh! Getting a kid in to see any kind of psychiatric provider is basically impossible*”.

Additionally, many immigrants need and prefer providers who speak their language and understand their way of life, adding another layer of complexity to accessing care. An informant explained that “*Another issue I can think of is probably [immigrants] are more comfortable seeing providers who speak Spanish, also understanding their culture, too*”.

### 3.4. Theme 3. Limited Access to Food That Can Support Healthy Dietary Patterns

Informants stated that because the grocery stores in these small towns have a limited food inventory, the residents rely on charitable giving and community sharing for their primary sources of food. In recent years, food distributions from bigger town food pantries have ceased or reduced in scale in these small towns as service areas have had to narrow their focus due to high demand and limited resources. These reduced distributions force residents to travel at least 15 miles to achieve healthy outcomes. As one informant stated, “*We don’t have a lot of grocers in town, and so when people have a need for groceries, I often see residents going out and making big trips and bringing it back for other residents and so kind of a community sharing in that sense*”.

The difficulty of acquiring food and/or groceries may be due to a lack of sidewalk infrastructure, human mobility issues, weather conditions, and extensive travel distances. The aging population is isolated from these resources because of fear of driving and lack of technology literacy. As one informant stated, “*You need to go get groceries and you don’t have a car. There’s an interstate over in the way*”. Another informant said, “*They (aging person) don’t drive anymore. They don’t have family (locally) here to take them to the store. They’re not comfortable using the technology to access food delivery options*”.

### 3.5. Theme 4. Inadequate Transportation Services

Lack of Transportation. The population density in small towns is often smaller compared to that in urban areas, correlating with limited transportation availability and options. Therefore, small towns are more likely to face certain transportation challenges regarding its availability, reliability, and affordability. Sparsely populated towns tend to be more prone to fewer public transportation services, fixed routes and times, a reduced access to private rideshare companies such as Uber and Lyft. The private rideshare services are also more expensive due to the limited availability. Additionally, aging residents may feel uncomfortable or nervous traveling into more urban areas, be unsure of how to use technology, and live on a fixed income. One informant said, “*They only know about Uber or think things are really expensive*”.

For those who do have a form of transportation, its use may be limited. For example, one informant noted that many families only have access to one car: “*They’re sharing a car, maybe one parent has a car. And so that makes it difficult for our families to get that some of those services*”. For others, they use bicycles or walk, as one informant stated: “*Several that (come to the food pantry) come on a bicycle or walk to the food pantry. Because they don’t have any kind of transportation*”.

Distances to services. Public transportation or community-based services are often needed to support those in need because those who do not own a vehicle or have a low income are more likely to lack transportation options. As the populations of popular geographic areas grow, the demand for healthcare increases, resulting in individuals in small towns navigating to bigger urban areas to access services. Due to a lack of services, small-town residents in Williamson County must travel more than 15 miles for healthcare services. An informant said, “*If* y*ou have to go in for tests, or whatever, you have to go into Georgetown. [And if you need specialty treatment], you have to go to Round Rock. [Aging people] are very nervous with the traffic*”.

As another informant reiterated, “*But [facilities in Georgetown, closest to them geographically] they’re not fully functioning hospitals, right? So, if there is a heart attack or even a snake bite, which is a super common thing out here, we’ve got to go to Round Rock right? We’ve got to go into Round Rock Baylor Scott & White, I think. You have to find whatever facility has the anti-venom that you need. And it takes travel [24 miles]*”.

### 3.6. Theme 5. Substance Use, Violence, and Crime

Crime, violence, and substance use are often overlooked due to the limited resources required to pay law enforcement and responding staff and limited prevention and education programs and resources. One informant explained how the shortage of local police and the flight of the local police workforce to surrounding larger cities affect crime: “*I mean, we’ve got a limited police staff. So, there’s a lot of folks that know when the police are on duty and when they’re not and so we have seen an uptick in crime, as we’ve seen our (police force) numbers dwindle, and police officers as they left for other opportunities (in bigger cities for more pay) and so we’ll see, you know, at night there’s more crime or more activity than we’d hoped for. But it’s because they’re aware that, you know there’s not a 24-h police Department*”. Specifically, informants expressed concerns about substance use. An informant said, “*There is just so much obviously marijuana, THC. I really worry about it. We haven’t had like a Fentanyl death, but I feel like I’m always waiting for it to happen*”.

### 3.7. Theme 6: Stressful Community Dynamics and an Environment That Influences Individual and Community Well-Being

Civic Participation. This study’s informants reported that their small towns lack the financial resources and public space required to host social events, like cafes, restaurants, or entertainment venues. The informants also explained that the existing facilities charge rental fees that are too high for non-profit organizations to pay. In addition, the informants mentioned that residents report not being aware of opportunities that are coordinated due to limited or poor communication and/or because they do not own the necessary devices or know how to use digital platforms. The informants mentioned that they are required to drive to bigger neighboring towns when they want to participate in community events. An informant said, “*The kids are so bored out there, there’s just like not a lot to do in our small towns, and that’s why they do all the sports activities, which is great. But they’re still bored*”. The informants explained why there is a lack of formal and structured social activities in which to participate in as a community. An informant said, “*We [our town] don’t have a beautiful square. And it is very much a truck stop and bedroom community*”.

Social Cohesion. The informants expressed a strong desire to move to and live in a small town to experience the small-town mindset and feeling of community unity. They reported craving a community that fosters and maintains close-knit relationships. They emphasized the value of managing their care, well-being, and personal connections within their community and their desire for it. As one informant shared, “*Because it’s small. There is a lot of genuine care and connection*”. As another informant reiterated, “*I would definitely say that the greatest strength here is the community itself, right? There is a small-town community*”.

However, the informants were concerned that a byproduct of the rapid population growth spreading into their small towns would be a decrease in social cohesion and the quality of their community’s heritage. The informants expressed fear that their small town might lose its small-town feel altogether and become a tourist destination. The informants also explained that they have witnessed their city leadership invest more in catering to tourists rather than the community members that live there.

Mistrust in Small-Town Leadership. There was a notable issue within these small communities wherein the informants expressed their concern with their predominantly white leaders, holding multiple responsibilities. Some of the informants reported leaders taking passive exclusionary measures, particularly with respect to residents whose personal beliefs and cultural backgrounds differ from a town’s primary systems. As one informant explained, “*You’re gonna see a similar representation to what is just across the country where there’s people in leadership, and people in teaching positions are predominantly white. We do have some diversity, but it’s definitely, not statistically representative*”. As another informant expressed, “*I will say that I do believe that it feels like a racial divide at times. And I feel minorities in small towns are targeted in some ways or that they’re not listened to. Many of our minority families don’t trust the school system*”.

Many of the informants reported that if a person is not originally from their small town, then it takes a while for the community to accept and trust them. As a result, churches are heavily relied upon as a trusted source of help and resources for community-based organizations, schools, and/or government entities. As one informant shared, “*So [city name] is the kind of town where there’s a very big distrust of outsiders. And so, a lot of things are handled informally. For example, there’s a lot of people who go to church for trusted information resources. So, food pantries and clothes closets are often in the churches. All these things are centered around churches and our people are gonna go to somebody that they already know. They’re not gonna trust some kind of like outside organization*”.

Some of the informants also explained that Latino parents express concern for their children due to a lack of diversity, teaching methods, and/or the structure of the education system. The informants reported that students from minority backgrounds often feel excluded, especially since the teaching staff are predominantly white and do not understand the culture and norms of the families they are serving. An informant reported that “*There’s a lot more stigma in those communities (minorities) around asking for help, needing help*”.

### 3.8. Theme 7. Financial Instability and Unreliable and Insufficient Infrastructure

Affordability and an inability to obtain insurance. People lacking adequate insurance coverage face significant health challenges. High co-pays and unstable incomes further limit access to necessary care. Medicaid inconsistencies and substandard housing exacerbate these issues, leading to toxic stress across generations. Some families even travel to other countries for affordable healthcare. As one informant said, “*A lot of what we see as the struggle is either people don’t have insurance, or their insurance doesn’t pay for a mental health component, or co-pays are just more than what they would want to pay or can pay. So, we do try to help them to connect but they can’t access that unless they have the money, yeah, generally, they’re not doing anything (for healthcare needs)*”.

Another informant reflected on a conversation with a client: “*They will go all the way to Mexico and go to dentists to get their healthcare needs met, like I asked them like, well, but you don’t go all the time. [They said] No, we go on Christmas break. We have 2 weeks off, so we’ll go during Christmas, and we all get our teeth cleaned, and we all get stocked up on our medications. Like they go down and get like all that they need and bring it back with them*”.

### 3.9. Theme 8. Community Strengths and Potential Resources

The informants described schools and churches as serving as a central resource for the community, providing essential services and cultivating a strong sense of connection and care among residents. One of the small towns has a community library offering various services and social activities for youths, families, and seniors. To foster a stronger community in these small towns, the informants reflected that they thought that it is important to raise awareness about local events and resources, celebrate cultural diversity, and build relationships across different ethnicities and cultures to enhance community unity. Effective communication through various channels and building trust, especially with immigrant families, can help achieve this goal. As one informant shared, “*But there really is this sense of community there. And there’s even in the school, right? Because the school really is sort of the center of the community. I would say that of the churches. So, there’s definitely a sense of like kids feel known. Kids don’t feel like they’re getting lost or falling through the cracks or anything like that. And I feel like that. There’s a sense of like trying to look out for each other*”.

Another informant said, “This cards group has kind of turned into a therapy group. Our peer support specialists seem to be with them a lot and visiting with them”. As this quote illustrates, small-town residents turn to social events for connection, providing opportunities for self-determination and health promotion.

## 4. Discussion

Health disparities in small towns stem from several interconnected challenges. Social determinants like low general education, coupled with low health literacy, often prevent residents from understanding which resources are available or knowing how to access them. There are small towns in Williamson County that are significantly more likely to have people living below the poverty level and have significantly lower median household incomes and significantly less likely to have a high school diploma or higher degree when compared to Williamson County as a whole [31]. United for Alice reported that in rural areas in Texas, 16% of households’ residents tend to live in poverty, which can be compared to 13% for their urban counterparts, and 31% of total households’ residents tend to live below the Asset, Limited, Income Constricted, Employed (ALICE) threshold, while this figure is 29% for urban residents [32]. Healthy Williamson County [33] further detailed that several small towns in Williamson County have more families living below the ALICE threshold than bigger cities in Williamson County. In fact, in the small towns we analyzed, 29–39% of all families lived below the ALICE threshold, which can be compared to the figure of only 24% for Williamson County. High rates of uninsured/underinsured individuals and financial constraints exacerbate these issues. Limited providers—including specialists—and healthcare facilities force residents to travel 15–30 miles, which sometimes prevents them from receiving care. Urban spillover increases demand for these services but also entices the already small workforce to seek employment in larger cities, where there are more resources. Additionally, mental health service providers and options are even scarcer, and the stigma associated with seeking such care in a small town can be greater than in larger urban areas. Nevertheless, many residents were adamant about emphasizing the importance and high value of their community’s strengths, mindset, and assets, and many also shared suggestions for solutions, which are incorporated into this discussion.

Addressing these challenges requires comprehensive strategies for improving access, infrastructure, and the healthcare workforce in small towns. Despite recent policies, programs, and payment models specifically developed for rural areas; simplified supervision of auxiliary behavioral health professionals; and the expansion of Medicare coverage and payment to include marriage and family therapists and mental health counselors, to name a few advances, significant health disparities still exist in the smaller towns in Williamson County, Texas [34]. This study helps highlight that small towns often do not benefit from federal, state, and/or county funding and programming resources.

Like residents of rural areas, small-town residents lag behind their urban counterparts in terms of both general education and health literacy. Combined with technological limitations, these factors may hinder rural Americans’ ability to navigate the healthcare system, limit their access to health information, and affect the quality of communication between providers and rural patients [35]. We argue that education and primary prevention are necessary to improve health literacy and health outcomes. Translating these findings back to the local level, including by increasing the number of community health workers working with local health departments, can help inform public health and primary care practitioners of how to prioritize individuals who are at risk and ultimately improve primary care for the 51% of the population with less-than-high health literacy [35]. Healthy People 2030 asserts that small-town residents and professionals would benefit from tailored health literacy programming. The National Action Plan to Improve Health Literacy [36] called for interventions that increase both organizational and personal health literacy, stating that, on the organizational health literacy front, goals should include promoting changes in the health care system that improve communication, informed decision-making, and access to culturally and linguistically appropriate health information and services. The authors agree with the National Action Plan’s aim of promoting accurate, standards-based, developmentally appropriate personal health literacy goals. The authors also advocate for health and science information and curricula to be dispersed in childcare and throughout the educational pipeline as well as expanded into English language instruction.

This study reveals factors that influence health care access in some small towns. Most importantly, there is often a shortage of all types of healthcare providers and facilities. The Centers for Disease Control and Prevention report shortages of primary care physicians, nurses, community health workers, pharmacists, paramedics, and/or physical/occupational therapists; and health care clinics, pharmacies, and hospitals are inaccessible due to their location [37]. Texas, which has a large rural population, is one of the ten states that elected to not expand Medicaid under the Affordable Care Act (ACA), which resulted in an uninsured rate of 21.7% of Texan adults aged 19 to 64, which is almost double the national rate, 11.0%, and 11.9% of children under the age 19 went without health insurance in 2023 in Texas, [38]. The ACA has been associated with access to a regular source of medical care and doctor visits in urban but not rural communities [39]. Since the literature indicates that rural America’s higher uninsured rate can be attributed to its having fewer large employers mandated to provide health insurance under the ACA, the same can be attributed to small towns. Many rural and small-town employers do not offer any insurance, and residents in these regions are more likely to participate in part-time, seasonal, or agricultural jobs without benefits [40]. To address these needs, we ask that healthcare funders begin prioritizing and allocating funds to build healthcare infrastructure and provide competitive salaries for providers working in small towns.

While telehealth may be thought to be a viable treatment option for meeting health care needs, research shows that limited coverage networks, low reimbursement rates, state licensure restrictions, and inadequate broadband access, among other wider policy changes, often hinder effective implementation in rural areas, small towns, and cities [41]. The residents from this study reflected on having inadequate space and staff with which to facilitate telehealth sessions, even if they were an option. We advocate for continuing to prioritize and increase incentives for the recruitment of rural providers. Although Texas remains among the top five states in the nation in terms of retaining physicians from undergraduate and graduate medical education, more physicians are needed [42]. Additionally, Flores et al. reported that only about 3% of medical residents in their last year of training indicated that they would want to practice medicine in communities of 25,000 people or less [42].

Healthy People 2030 emphasized that having access to healthy, safe, and affordable food is crucial to allow an individual to achieve a healthy dietary pattern. The small-town informants in this study repeatedly reported having limited access to affordable food options. Limited-access areas are defined as low-income areas that are further than one mile from a large grocery store or supermarket. The informants also consistently reported concern regarding the families they serve not having access to a personal vehicle or affordable and/or convenient public transportation for accessing healthy food [43]. The Central Texas Foodbank reported that there are 6.2 times as many convenience stores and fast-food restaurants as there are grocery stores in Central Texas. These trends indicate higher availability of ultra-processed foods and limited availability of fresh, nutritious foods [44]. Also compounding the problem, small towns tend to lack access to most types of food retail outlets or other types of grocery stores that have been approved by the U.S. Department of Agriculture to accept support from the Supplemental Nutrition Assistance Program (SNAP), including convenience stores, supermarkets, and farmers markets [45].

The Williamson County and Cities Health District reported that households with children receiving SNAP benefits (a federal program that provides low-income families with electronic benefit transfers that can be used to purchase adequate and nutritious food) were more likely to be living in bigger cities, not in small towns. This may be attributed to many factors, such as they are not aware of resources, an inability to access these resources, and the stigma attached to receiving public assistance [46]. According to Healthy Williamson County, 58% of Williamson County children are food-insecure and likely ineligible for assistance; this can be compared to the Texas value of 35% and the U.S. value of 30%. This is an increase from its prior value of 54% in 2021 [47]. All these variables together imply a direct risk for members of low-income families in small towns who develop chronic diseases such as obesity and diabetes [48].

Public transportation in rural areas is both scarcely available and rarely used; only 3% of rural residents regularly use public transit, which can be compared to 21% for urban residents [49]. In many U.S. counties, older adults, those with disabilities, and individuals in low-income communities lack access to adequate and affordable public transportation [50]. Additionally, research has shown that across the whole U.S., the average distance traveled for medical/dental care is 10.2 miles, with rural trips averaging 17.5 miles, while the distance is 8.3 miles for urban residents [51]. The residents in our study emphasized the importance of adequate transportation services with respect to navigation, independence, and travel. Previous evidence suggests that collaborating with transportation services or companies would help increase routes of operation and thus decrease the demand or need in small towns [52]. Our findings indicated that there is a great need for services, especially for those who are the most vulnerable and distant from services and resources.

Crime, violence, and substance use further impact community well-being. The limited law enforcement presence in small towns leaves vulnerable groups feeling unsafe, and substance use issues, particularly concerning marijuana and the looming threat of fentanyl, are significant concerns due to the lack of resources and prevention programs. Kelling et al. highlighted the importance of improving data quality for policing in towns with fewer than 50,000 residents, recommending the involvement of diverse stakeholders, including policymakers, researchers, and community members, in the process [53]. Similarly, rural adults have higher rates of tobacco use and other substance use than their urban counterparts for multiple reasons, such as limited resources for prevention, treatment, and recovery [54]. We call for further research to better understand and address these challenges in small-town contexts.

The residents interviewed in this study repeatedly expressed concern regarding the lack of opportunities to participate in civic and social activities directly in their small towns. There are several reasons why small towns may offer limited social engagement experiences. For example, rural communities tend not to promote as many culturally diverse events or have as many vocationally diverse role models compared to urban communities [55]. Additionally, rural schools tend to lack the infrastructure and human resources needed for developing assorted school–community partnerships [56].

Researchers suggest neighboring communities come together to support small towns. It is imperative for small-town leadership to focus on securing sustainable strategies to ensure residents have access to social and community-building opportunities. Cavaye suggests that informal, temporary, and social ways of participating in communities are effective for community engagement [57].

Many residents cherish the close-knit relationships and support found within their communities. It is imperative for small-town leadership to find ways to maintain their small-town-community feeling while intentionally growing their infrastructure to support population growth. Further research is needed to better understand how social cohesion affects health as well as how it can be used to reduce health disparities. This evidence will facilitate public health efforts to address social cohesion as a social determinant of health.

The informants analyzed in this study counterargued that small-town dynamics and a sense of unity were often unbalanced by mistrust in leadership. And, often due to local leaders holding multiple responsibilities and the dominance of church-based systems, this could be very detrimental to “outsiders’” sense of belonging. To address these issues, we argue that there is a need to increase transparency and inclusivity within small towns, as well as create more leadership training/programs, which are designed to broaden perspectives and foster collaboration across diverse community groups. More disturbingly, this mistrust extends to and exists in the school system, where Latino and African American families feel excluded due to a lack of diversity and inclusion. Research shows that the way schools handle diversity has long-term implications, particularly for students’ health outcomes and health disparities [58]. We suggest implementing initiatives focused on promoting the value of diversity in schools, such as integrating multicultural education into curricula and actively acknowledging the diverse backgrounds of students.

Healthcare affordability also affects one’s ability to access healthcare. Rural Americans are poorer than their urban counterparts and thus more likely to report delaying care, or not obtaining prescription medications, due to cost [59]. Although some initiatives, such as community-directed interventions, mobile clinics, or Medicaid and Medicare, help bring healthcare to small-town residents and are designed to improve healthcare access and further reduce the disparities, many programs rely on inconsistent funding sources, which can lead to interruptions in services. Many rural residents who qualify for Medicaid or Medicare do not obtain these benefits due to several barriers. These include a lack of awareness about their eligibility, the complexity of the application process, and the stigma associated with receiving government assistance. Additionally, access issues, administrative hurdles, and misinformation about the programs further complicate enrollment [60,61]. Addressing these challenges requires improved outreach, simplified application procedures, and better support to help eligible individuals navigate the system. Johnston et al. recommend providing free, unbiased navigational support through independent nonprofits with salaried navigators. Strengthening the State Health Insurance Assistance Program (SHIP) by expanding its reach, ensuring counselors are paid and well-trained, and increasing federal funding are recommended [61].

Countering social ailments experienced in small-town communities, our study shows that the school acts as a vital hub, offering essential services and fostering community connection. Moreover, social activities for seniors, a community library, and active local churches further support and unite residents. These are considered to be communities’ assets that could strengthen community bonds. There is a substantial amount of evidence about health disparities and the social determinants of health in rural areas; however, to our knowledge, this study is one of the first to examine them in small towns that do not qualify as rural according to standard definitions. Additional data are needed to better understand the internal dynamics and needs of small towns in order to continue addressing disparities in health outcomes. Successful strategic planning for small towns involves engaging the community in creating a shared vision, versifying the local economy, and supporting local businesses and cultural activities to strengthen community identity [62]. Developing multi-use facilities maximizes resource utilization, while focusing on sustainable practices ensures long-term health and resilience [62]. These strategies not only foster economic growth but also enhance the quality of life for residents by promoting a cohesive, vibrant, and sustainable community.

## 5. Actionable Recommendations

By prioritizing these actionable steps, funders, lawmakers, and city administrators can contribute to building stronger, healthier, and more resilient small-town communities, ultimately improving health equity and quality of life for all residents.

System-level change recommendations:

- Standardize the definitions of urban and rural to ensure equitable access to funding and resources.
- Acknowledge infrastructure deficits in small towns as a public-policy issue contributing to health and social inequities.
- Integrate health literacy into the educational pipeline, from early childhood through higher education.
- Integrate diversity and inclusion initiatives into school curricula and community programs to foster understanding and empathy.
- Ensure that geographically isolated and small towns are included in public health data collection and improvement efforts.
- Increase partnerships with academic institutions to conduct research focused on community development, sustainability, and quality of life in small towns.
- Investigate the role of social cohesion as a determinant of health and its impact on community well-being.

Focused funding for improving accessibility for small towns:

- Increase funding for healthcare infrastructure, community development, and the social determinants of health, especially in health equity zones.
- Simplify grant and funding application processes.
- Allow flexibility in funding to encourage community-driven solutions and compensate residents for their input.
- Offer incentives, such as competitive salaries, to healthcare providers working in rural areas.
- Prioritize funding for mobile healthcare solutions, including clinics, food delivery, and community engagement activities.
- Facilitate collaboration among city, county, and state officials to improve public transportation and access to resources across rural and urban areas.
- Healthcare systems should fund internal transportation services for low-income, fixed income, and disabled populations.

Develop sustainable community resource hubs:

- Establish permanent community resource hubs with full-time community health workers to help residents navigate available resources.
- Increase employment among community health workers and peer specialists to help vulnerable populations navigate services.
- Offer no-cost training in health literacy, cultural humility, and trauma-informed care for community leaders and organizations.
- Encourage cultural humility training for community leaders, healthcare providers, and local organizations.

## 6. Limitations

Some limitations of this study need to be noted. This study’s participants represent a convenience sample of key partners from small towns in Williamson County, Texas. Moreover, the participants were all females, and half of them were white. Therefore, the themes may not represent the full diversity (e.g., regarding ethnic, provider specialty, gender, and socioeconomic class) of other small cities and towns. Further studies are needed to establish a broader list of themes using these and other demographics to enhance the generality of the results. While this study offers important insights, its results cannot be generalized outside the small-town setting. Qualitative methodologies have inherent limitations, such as the possibility of a subjective tendency due to selection bias. Future quantitative investigations could enrich the findings of this study. Moreover, the cross-sectional nature of this study limits the ability to determine the cause–effect relationships between the environment and residents’ overall wellbeing. Longitudinal studies are needed to understand the long-term impacts on these phenomena.

## 7. Conclusions

In conclusion, the health disparities faced by small towns are a multifaceted issue, deeply rooted in social determinants, access barriers, and systemic challenges. While recent policies and initiatives aimed at improving healthcare access in rural areas represent steps in the right direction, significant gaps remain, particularly in small towns like those in Williamson County, Texas, that do not qualify as “rural” by federal standards. The interplay of low health literacy, limited resources, inadequate transportation, and financial instability compounds these challenges, often leaving residents without essential care.

However, this study also highlights the strengths within these communities, such as organized collaboratives, schools as hubs of support, and the close-knit relationships that foster social cohesion. Future recommendations include employing a place-based approach study design that leverages local knowledge, assets, and collaborative governance. Place-based research is vital in building culture and making important insights more visible to diverse audiences. Community partners are more likely to succeed in increasing health literacy, improving infrastructure, and ultimately reducing disparities if they can leverage research-based strategies tailored to address economic, social, and territorial inequalities [63]. Comprehensive strategies that engage community members and regional leadership are essential for creating a sustainable, health-focused environment in small towns. More studies focusing on small towns, particularly those burdened by urban spillover, and healthcare disparities are needed to provide valuable insights into community development, sustainability, and quality of life.

## Figures and Tables

**Table 1:** The formal outline used to facilitate interviews with the participants.

Research Question	Open-Ended Questions
How do critical health issues specifically impact low-income, underserved, or uninsured populations?	1.1 Who do the top health issues affect the most (e.g., age groups, racial and ethnic groups, socioeconomic groups, and geographic subsets)?
	1.2 How do critical health (medical, dental, and mental) issues specifically impact low-income, underserved, or uninsured populations in your city?
	1.3 What are the things that make your city feel unsafe, unwelcoming, or inaccessible?
2. What are some of the community’s greatest strengths and assets?	2.1 What are some of the community’s greatest strengths and assets?
	2.2 How could your community’s strengths be leveraged to address the health issues identified?
	2.3 What are your suggestions for ways to engage and build trust with community members, particularly low-income, underserved populations; ethnic and racial minorities; and less-English-proficient residents, to address the critical health issues of this county?

**Table 2:** Descriptive summary of interviewees (N = 8).

Demographic Characteristic	*n*	%
Age		
30–45 years	4	50.0
46–60 years	3	37.5
≥60 years	1	12.5
Gender		
Female	8	100.0
Race/Ethnicity		
Hispanic White	2	25.0
White	4	50.0
Mixed races	1	12.5
City		
Bartlett	4	50.0
Granger	1	12.5
Jarrell	1	12.5
Thrall	1	12.5
Position types		
School-based program coordinator (nurse, counselor, and therapist)	4	50.0
City staff	1	25.0
Emergency management staff	1	25.0
Food pantry staff	1	25.0
Library and resource hub staff	1	25.0

**Table 3:** Codes used in qualitative analysis related to themes.

Theme	Categories	# of Participants	% of Participants (N = 8)	Number of Quotes
Low levels of community and professional health literacy and a lack of healthcare navigation support in schools and community-based support systems	Systematic issues regarding community-based services and schools	6	75%	13
	Lack of health literacy, expertise, timing, and training	7	88%	17
Limited funding forhealthcare services and limited capacity to support access to them	Limitations regarding funding and sustainability	7	88%	14
	Healthcare provider shortage	7	88%	28
Limited access to food that can support healthy dietary patterns	Limited access to food	4	50%	7
Inadequate transportation services	Lack of transportation	8	100%	28
	Distance to services	8	100%	20
Substance use, violence, and crime	Substance use, violence, and crime	5	63%	13
Stressful community dynamics and an environment that negatively influences individual and community well-being	Civic participation	4	50%	5
	Social cohesion	3	38%	9
	Mistrust in leadership	4	50%	10
Financial instability and unreliable and insufficient infrastructure	Affordability	7	88%	17
	Affordability and inability to obtain insurance	6	75%	13
Community strengths and potential resources	Small-town feel	6	75%	24
	Community unity	4	50%	8

## Data Availability

Data are not available for sharing because a data-sharing plan was not included in the project protocol in the IRB review.
